# Hardware-software co-design of an open-source automatic multimodal whole slide histopathology imaging system

**DOI:** 10.1117/1.JBO.28.2.026501

**Published:** 2023-02-08

**Authors:** Bin Li, Michael S. Nelson, Jenu V. Chacko, Nathan Cudworth, Kevin W. Eliceiri

**Affiliations:** a University of Wisconsin–Madison, Center for Quantitative Cell Imaging, Madison, Wisconsin, United States; b University of Wisconsin–Madison, Department of Biomedical Engineering, Madison, Wisconsin, United States; c Morgridge Institute for Research, Madison, Wisconsin, United States; d University of Wisconsin–Madison, Department of Medical Physics, Madison, Wisconsin, United States

**Keywords:** whole slide imaging, histopathology, multimodal imaging, second harmonic generation, neural networks, workflows

## Abstract

**Significance:**

Advanced digital control of microscopes and programmable data acquisition workflows have become increasingly important for improving the throughput and reproducibility of optical imaging experiments. Combinations of imaging modalities have enabled a more comprehensive understanding of tissue biology and tumor microenvironments in histopathological studies. However, insufficient imaging throughput and complicated workflows still limit the scalability of multimodal histopathology imaging.

**Aim:**

We present a hardware-software co-design of a whole slide scanning system for high-throughput multimodal tissue imaging, including brightfield (BF) and laser scanning microscopy.

**Approach:**

The system can automatically detect regions of interest using deep neural networks in a low-magnification rapid BF scan of the tissue slide and then conduct high-resolution BF scanning and laser scanning imaging on targeted regions with deep learning-based run-time denoising and resolution enhancement. The acquisition workflow is built using Pycro-Manager, a Python package that bridges hardware control libraries of the Java-based open-source microscopy software Micro-Manager in a Python environment.

**Results:**

The system can achieve optimized imaging settings for both modalities with minimized human intervention and speed up the laser scanning by an order of magnitude with run-time image processing.

**Conclusions:**

The system integrates the acquisition pipeline and data analysis pipeline into a single workflow that improves the throughput and reproducibility of multimodal histopathological imaging.

## 1 Introduction

Technological advances in microscope automation and optics have led to the emergence of computer-controlled motorized microscopes that can accomplish complex data acquisitions with little human intervention. Many of the advances have been utilized in histopathology, the gold standard for the assessment of disease. The development of digital slide scanners and whole slide image (WSI) scanning techniques has enabled conventional hematoxylin and eosin (H&E)-stained glass slides to be converted into digital images with microscopic resolutions for diagnosis, research, and medical education.^1^^,^^2^ Consequently, digital pathology has become a popular research area in which innovation is sought in the analysis of digital images and the development of computational instruments to increase diagnostic accuracy and imaging throughput.^3^^,^^4^ The attention has further increased due to the success of deep neural networks (DNNs) in image analysis tasks such as image classification, segmentation, and image restoration.^5^^,^^6^

Although in hospitals most slides are stained with H&E and viewed under a brightfield (BF) microscope, advances in optics have greatly diversified the ways tissues can be examined in research labs. Techniques such as fluorescence microscopy,^7^ multiphoton microscopy,^8^^,^^9^ polarization and phase microscopy,^10^^–^^13^ and chemical imaging^14^^,^^15^ have become increasingly popular in tissue research because of their ability to provide additional information and context about cells and their environment. Scientific findings from research laboratories have been applied to solve clinical problems, with many of them proving to have diagnostic and prognostic applications. For example, tumor-associated collagen signatures,^16^^,^^17^ a negatively prognostic biomarker defined by alterations in collagen orientation and deposition during tumor progression, were discovered using second-harmonic generation microscopy (SHG).^9^ More complex optical modalities, such as fluorescence lifetime microscopy (FLIM) and Raman spectroscopy, have also emerged as powerful tools for studying cancer metabolism.^14^^,^^18^^,^^19^

Despite the popularity of multimodal histopathology studies in research, the scales of these studies are often limited due to the high complexity and/or cost of the imaging instruments and the low throughput of the imaging experiments. Performing multimodal imaging can be time-consuming for whole tissue sections at high image resolution. This scaling problem is especially problematic for modalities that involve point scanning and hardware parameter tuning. For example, scanning a whole tissue section using SHG at a resolution necessary to provide collagen fiber information could easily take several days to complete, all while needing to maintain the instrument’s focus on the tissue sample. Reducing the complexity and increasing the throughput of multimodal imaging experiments for histopathology remains an open challenge.^20^^–^^22^

From the advancements in open-source software developed for bioimage acquisition and analysis, image acquisition and analysis workflows can now be automated using scripts and executed efficiently to allow for batch acquisitions. For histopathological image analysis, tools such as QuPath,^23^ Cytomine,^24^ Histomicstk,^25^ Fiji/ImageJ,^26^ and CellProfiler^27^ are frequently used for image visualization, segmentation, and cell quantification. To integrate run-time analysis into the acquisition workflow, the open-source microscope control platform Micro-Manager^28^ provides an interface to call Fiji/ImageJ during the acquisition, which provides a basis for building integrated acquisition-analysis workflows. However, Fiji/ImageJ is not designed to handle pyramidal WSI (which can have dimensions up to 100,000×100,000  pixels) and leads to suboptimal performance or even failure to load the images at all. Moreover, workflows scripted in the ImageJ-Micro-Manager environment are often limited to simple workflows with insufficient flexibility, such as the lack of machine learning libraries and access to graphics processing units. Integration of the latest tools and models in computer vision and machine learning, such as DNNs, remains challenging.

Leveraging machine learning in microscopy automation is a promising solution that enables the design of sophisticated imaging experiments and improve experimental throughput. Conrad et al.^29^ developed a LabVIEW-based intelligent acquisition system empowered by a machine learning model that automatically triggered high-resolution (HR) scans based on cell events, such as mitosis, detected in low-resolution (LR) screening. This automation minimized the time-intensive work that the researchers otherwise have had to spend on low-level observations and allowed them to concentrate on high-level experiment design and data analysis. Complex and flexible acquisition workflows with run-time analysis capabilities can also be achieved in the Konstanz Information Miner (KNIME),^30^ a powerful analytic platform that enables the integration of image analysis and microscope control in a closed loop. However, both LabVIEW and KNIME lack both robust support for the large variety of microscopy devices available and the ability to handle WSI datasets. The recently developed open-source Python package Pycro-Manager^31^ allows for complex acquisition and analysis pipelines that involve microscope control, data acquisition, and data analysis to be programmed in a single Python environment. Moreover, acquisitions with run-time analysis and feedback loops can be built and customized, utilizing the power of a wide variety of data analysis Python packages, including popular deep learning (DL) platforms, such as PyTorch and Tensorflow. In addition, platforms developed for handling and/or analyzing WSI, such as OpenSlide^32^ and QuPath,^23^ could be utilized in Python via their Python or command line interfaces. Combining the flexibility of the Python programming environment and the ability to access Micro-Manager Java libraries make Pycro-Manager a suitable tool for building an open-source automatic multimodal histopathological imaging system.

In this paper, we present a hardware-software co-designed imaging system for high-throughput multimodal histopathology. The optical path is designed with two coupled light paths for BF and laser scanning microscopy (LSM) that allows for switching between these two modalities using a simple shutter control. The system is equipped with a three-axis motorized stage, a motorized condenser, and a motorized dual objective slider. The motorized components can be adjusted via the software to form optimal configurations for different combinations of magnifications and modalities. The acquisition programs of our system are written in Python using Pycro-Manager, presented in Jupyter Notebook. Our system has a trainable DL-based detection model that automatically detects targeted regions in a low-magnification rapid scan and switches to higher magnification for BF or LSM acquisition at targeted regions. Annotations can also be made manually and imported from QuPath,^23^ where the region of interests (ROIs) can be marked and translated to the stage coordinate system. During acquisition, in addition to standard run-time image correction algorithms, such as software autofocus and white balance, DL-based image restoration models can be applied to LSM images, including denoising and resolution enhancement. Evaluation of the DL-based image restoration methods on a pancreatic tissue microarray (TMA) slides set shows that the denoising and resolution enhancement model can improve the image signal-to-noise ratio (SNR) and image resolution, leading to shorter scan times while maintaining the necessary image quality for downstream image analysis. With selective acquisitions and run-time image restoration integrated into a single acquisition-analysis workflow, the system can considerably improve the throughput and repeatability of multimodal imaging for histopathology.

## 2 Methods

**Fig. 1:**
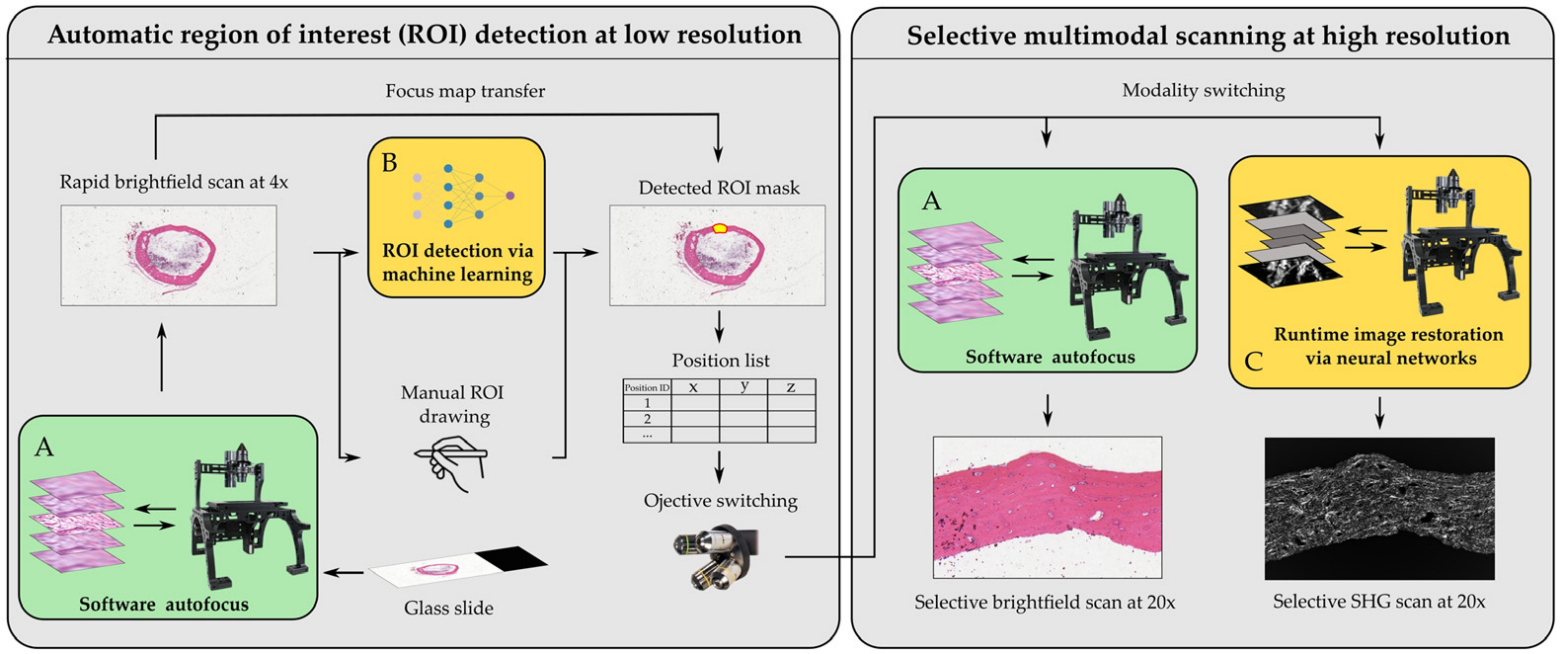
Overall workflow for multimodal imaging of tissue sections (SHG and BF). The annotated or detected region on a low-magnification rapid scan is imaged with SHG and BF at higher magnification. (A) Software autofocus module for BF scanning. The focus map is recorded during scanning and is used for high-magnification scanning or SHG scanning. (B) Automatic ROI detection using trained neural networks. The detection map is converted into a position list for selective acquisition. (C) Run-time image enhancement for SHG scanning using neural networks. Image resolution enhancement and denoising are performed on-the-fly during scanning.

The system consists of three major components; a Pycro-Manager-based acquisition program written in Jupyter Notebook, a SHG-BF coupled microscopy system, and DL-based image analysis modules built using PyTorch. The overall workflow is illustrated in Fig. 1. Once the sample is mounted on the stage, the system first captures a BF scan of the whole slide area at low magnification and produces a stitched pyramidal OME.TIFF file.^33^ Annotations can then be generated in two ways: 1) manual annotations are made in QuPath (v0.3.2) and exported as lists of coordinates within a CSV file or 2) Annotations are automatically generated by a DL model and exported as coordinate files. The coordinates files are then passed to the acquisition program and converted into position lists for selective acquisitions at high magnification, first for BF including an autofocus step and then for LSM. During the LSM acquisition, two types of DL-based image enhancement models, self-supervised denoising (SSD) and single-image super-resolution (SISR), are enabled to improve the apparent quality of the scanning results. The system will be explained in more detail in the following sections.

### 2.1 Software Structure and Acquisition Workflow

The acquisition program is built on Pycro-Manager (v0.18.1). Pycro-Manager is a Python package that communicates with Micro-Manager, which handles hardware control via Micro-Manager’s device adaptors, such as the laser scanning module OpenScan. The fast data transfer and translation layer between Python and Java in Pycro-Manager allows Java libraries to be called as if they were in Python. Because Micro-Manager already supports a large variety of microscopy hardware, integrating Pycro-Manager allows for customized acquisitions to be programmed in Python, enabling analytical Python packages to be easily inserted into the acquisition pipeline in a single Python environment.

The acquisition program is written in Jupyter Notebook. The program first deploys a fast prescan at low magnification (e.g., 4×). A position list covering the whole slide area is automatically generated according to the slide size, field of view size, and image pixel size. After the acquisition, the tiles are stitched according to the position list using the grid/collection plugin^34^ from ImageJ^26^ accessed from Python via PyImageJ. During the acquisition, software autofocus is applied (see Appendix B for details of the autofocus algorithm), and the focus position is recorded for each position. This is necessary because a tissue section mounted on a glass slide can have variations in terrain heights, which leads to varying focal planes at different locations. Background tiles are automatically recorded and averaged to produce a background image, which is used for white balancing and illumination correction. This scan can be done relatively quickly because of the large field of view (FOV) of each frame at low magnification. A 4× magnification objective is used for the low-magnification scan because it provides a good balance between the resolution of details needed to identify some tissue features and a wide FOV. The stitched prescan image is then exported in a BioFormats compatible pyramidal format (OME TIFF) with slide metadata (e.g., XY pixel size, objective magnification) by calling QuPath via its command line interface from Jupyter Notebook.

**Fig. 2:**
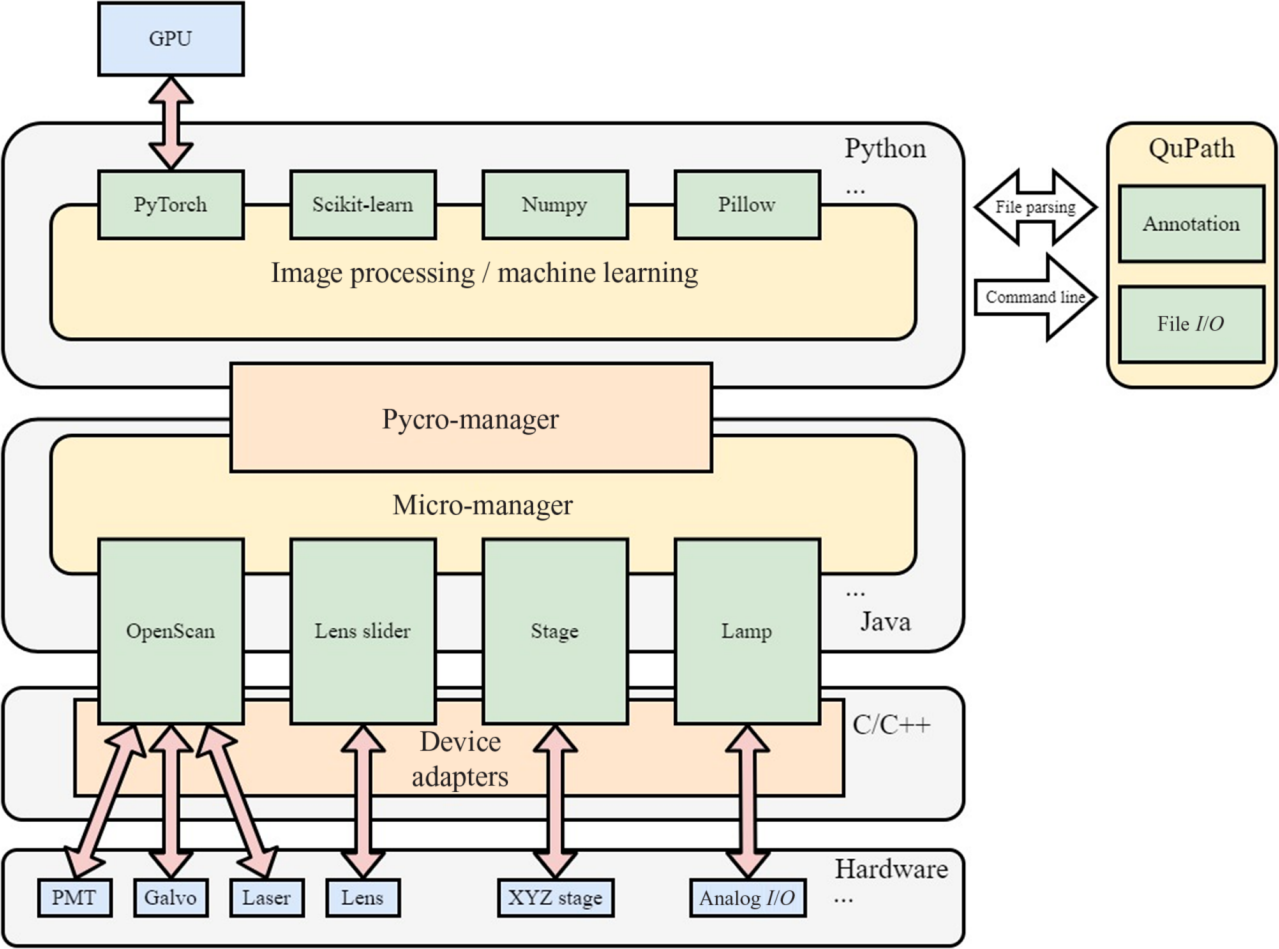
Architecture of the application. The acquisition hardware is controlled via Pycro-Manager, which interacts with Micro-manager and translates the Java libraries into Python. QuPath is called for annotation handling and reading/outputting annotation files.

The user can then make annotations by opening the low-magnification prescan image in QuPath. A QuPath script is used to generate acquisition tiles, shown in an overlay on the image, covering the annotated areas according to the FOV size at 20× magnification for BF and LSM. Alternatively, the annotations can be generated automatically by DL-based detection models (details covered in the following section). The acquisition program then uses the annotation(s) to perform a selective acquisition at a higher magnification with BF and LSM using the positions saved in the files. The LSM control is handled by an in-house software-OpenScan,^35^ a Micro-Manager device library that handles the generation of scanning waveforms, the control of scanning galvanometer, the scanning resolution, and the photomultiplier tube (PMT). The interactions of the software used in our system are illustrated in Fig. 2.

The focus of each tile at 20× BF is first roughly determined by interpolating the focus map recorded in the 4× prescan and then fine-tuned using the same autofocus algorithm with a narrower searching range during the 20× BF acquisition. The focus of each tile is again recorded and the focus map is interpolated and sampled to generate a more finely detailed focus map to be used for the LSM modality (the 20× LSM and BF FOV sizes can be slightly different, and there will be a Z offset between modalities). For multiphoton LSM or SHG, a z-stack is often beneficial due to the optical sectioning property of multiphoton microscopy resulting in incomplete coverage of the full depth of the tissue.^9^ The step size and range of the z-stack can be configured according to the sample thickness; the following experiments used a 5-μm step size with three steps. With the center of the tissue at each location roughly determined in the 20× BF scan, the number of slices in the z-stack of LSM can be reduced such that it only covers the depth around the center of the tissue at each location.

### 2.2 Multimodal Imaging System

The imaging system is designed to couple BF and multiphoton LSM light paths with the ability to switch between the two modalities via simple shutter control. A Tsunami Ti:Sapphire laser (Spectra-Physics, Santa Clara, California) tuned to 800 nm, with a pulse length of ∼100  fs, is directed through a Pockels cell (ConOptics, Danbury, Connecticut), half and quarter waveplates (ThorLabs, Newton, New Jersey), a beam expander (ThorLabs), a 3-mm galvanometer driven mirror pair (Cambridge, Bedford, Massachusetts), a scan (f=75  mm) and tube (f=250  mm) lens pair (ThorLabs), and a dichroic mirror (Semrock, Rochester, New York) and is focused by a 20×/0.75 NA air objective (Nikon, Melville, New York). SHG light is collected in the forward direction with a variable NA condenser (Olympus, Lombard, Illinois) and filtered with a 680-nm short-pass filter (Semrock) and an interference filter centered at 400 nm with a full-width at half-maximum bandwidth of 10 nm (ThorLabs). The back aperture of the condenser lens is focused onto a H7422-40P GaAsP PMT (Hamamatsu, Hamamatsu, Japan) using a secondary collection lens (f=150  mm, ThorLabs). The signal from the PMT is amplified with a C7319 integrating amplifier (Hamamatsu) and sampled with an analog data acquisition device NI PIXe-6356 (National Instruments, Austin, Texas). The galvanometer is controlled by an analog data output device NI PXIe-6738 (National Instruments, Austin, Texas). Timing among the galvo scanners, signal acquisition, and motorized stage positioning is achieved using our custom software called OpenScan in conjunction with the open-source software Micro-Manager (v2.0).^28^

**Fig. 3:**
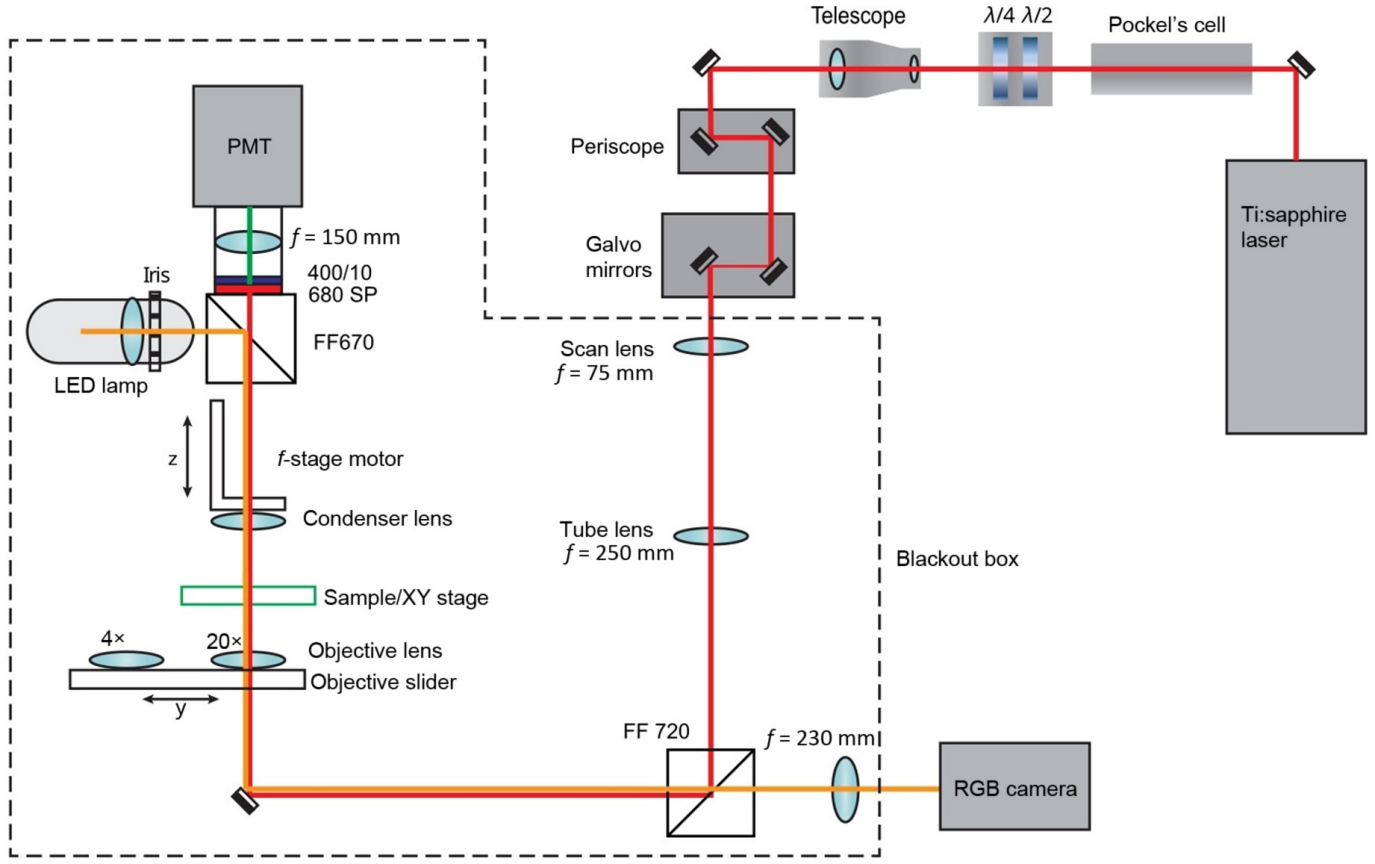
Optical schematic of the laser and BF light path. The 800-nm laser light is passed through an electro-optic modulator (Pockels Cell) and a half-wave plate followed by a quarter-wave plate (λ/2, λ/4). The polarized light is focused by a 75-mm focal length scan lens and a 250-mm focal length tube lens to the back aperture of a Nikon 20×/0.75 NA objective lens. Light from the sample is collected by an adjustable NA condenser lens and passed through a 670-nm dichroic mirror (FF670), a 680-nm short-pass filter (680 SP), and a 400-nm interference filter (400/10) and is focused by a 150-mm focal length collection lens to a Hamamatsu 7422-40P photomultiplier tube (PMT). White light is generated by an LED lamp and passed through the condenser and the sample and collected by either a Nikon 4×/0.13 NA objective or the 20× objective. The light passes through a 720-nm dichroic mirror (FF 720) and is focused by a 230-mm focal length tube lens onto a Qimaging QICAM Fast 1394 camera (RGB Camera).

The Rapid Automated Modular Microscope system (Applied Scientific Instrumentation, Eugene, Oregon) serves as our microscope base, and ASI motorized translation stages are used for x, y, and z motion control. BF images are captured with the same system using a MCWHL2 white LED lamp (ThorLabs) and both the 20×/0.75 NA objective and a 4×/0.13 NA objective (Nikon). White light from this lamp travels through the condenser directed by a short-pass dichroic mirror with a cutoff at 670 nm (Semrock). The white light is passed through the first dichroic, focused on an RGB camera (QICAM Fast 1394, Qimaging, Surrey, BC, Canada) with a collection lens (f=230  mm, ThorLabs). The image data transfer is handled by OpenScan/Micro-Manager. An objective slider (ASI) is used for easily switching between 4× and 20× objectives and a z-oriented motorized arm (f-stage, ASI) for optimizing the amount of light collected by the condenser for each imaging modality and objective position. All components of the laser light focusing and collection are contained in a blackout box as shown by the dashed line in Fig. 3.

### 2.3 Automatic ROIs Detection with Deep Neural Networks

In addition to acquiring annotated regions defined by user-entered annotations, our system can generate annotations automatically via DL-based detection models.

Convolutional neural networks (CNNs) have demonstrated state-of-the-art performance in many vision tasks and have been extensively used in computational histopathology for tasks such as tumor detection, gland segmentation, cell segmentation, and cell classification.^36^^–^^39^ A CNN differs from traditional machine learning methods in that the feature extractor is parameterized by layers of convolutions with learnable filters and nonlinear functions. The parameters of the filters are learned during the training, whereas most of the traditional machine learning models for vision tasks use handcrafted features, such as SIFT.^40^ With a sufficient amount of training data available, CNNs usually outperform traditional machine learning models and show better generalizability to unseen data. In our design, we use ResNet^41^ as the CNN backbone for building the detector. ResNet uses residual connections that allow for more convolution layers to be used in the network without suffering from gradient vanishing, and it can alleviate overfitting by providing “shortcuts” for certain information to skip redundant convolutional layers. The training settings can be found in Appendix A.1 The trained CNN are enabled in the acquisition workflow. The low-magnification scan is processed by the CNN, and the coordinates of positive areas are generated and saved in a position list. The position list is then used by the acquisition program for high magnification BF and SHG imaging.

### 2.4 Deep Learning-Based Run-Time Image Restoration

Performing laser scanning imaging, such as SHG, can be time-consuming due to the point-wise scanning only collecting a single pixel at a time. The imaging time is further extended if a z-stack is required to cover the sample thickness. A complete scan of a tissue section could take several days to complete. Thus solutions that enable faster scanning while maintaining image quality are desirable. The image quality is mainly determined by the SNR and scanning resolution. Generally, the SNR of LSM is proportional to the square root of the number of photons (which is proportional to the dwelling time of each scanning point). The scanning resolution of the images is determined by the number of scanning points along each dimension (apart from optical resolution determined by the NA of the objective). Ideally, the scanning resolution should match the optical resolution such that Nyquist sampling for the optical signals is met. This means that a lower scanning resolution might undersample the optical signals and a higher scanning resolution, i.e., more scanning points along each dimension, would be necessary to increase the image resolution. Due to LSM deploying point-to-point scanning, the scanning time increases quadratically with the scanning resolution. Fortunately, recent advances in DL-based image restoration and enhancement techniques have brought new solutions to address this problem. In our system, two types of DL-based image enhancement models are implemented to shorten the scanning time while preserving the image quality, including an SSD model and a SISR model. With a successfully trained model enabled during the acquisition, the scanning procedures can be performed an order of magnitude faster while maintaining image quality comparable to that of the original scan.

#### 2.4.1 Self-supervised denoising model

The signals that form an image are considered to be x=s+n, where s and n are drawn from two the joint distributions:

p(s,n)=p(s)p(n|s). (1)

Within a given radius R of i, the signal value $s_{i}$ conditionally depends on the observation of signals at other locations within the radius (i.e., $p(s_{i})\neq p(s_{i}|s_{j})$ s. t. $\left\{s_{j}\in R,j\neq i\right\}$) but is independent of the noise at other locations (i.e., $p(s_{i})=p(s_{i}|n_{j})$ s. t. $\left\{n_{j}\in R,j\neq i\right\}$), meaning that the distribution of the noise conditioning on the signal factorizes as

$$p(n|s)=\underset{i}{\prod }p(n_{i}|s_{i}).$$ (2)

Under these assumptions, the expectation of the pixel value at i is estimated by a blind spot network that observes the surrounding region of the pixel i.^42^ Because $E[x_{i}]=E[s_{i}]+E[n_{i}]=E[s_{i}]$ (assuming the noise is zero mean, i.e., $E[n_{i}]=0$), the estimation is free of noise.^42^ This suggests that the model can estimate the clean image even without observing clean images in the training phase. Such methods are known as self-supervised learning (SSl) denoising.^43^ Models such as Noise2Void have been successfully deployed for denoising microscopic data and tested for the cases of Poisson noise and Gaussian noise.^42^

**Fig. 4:**
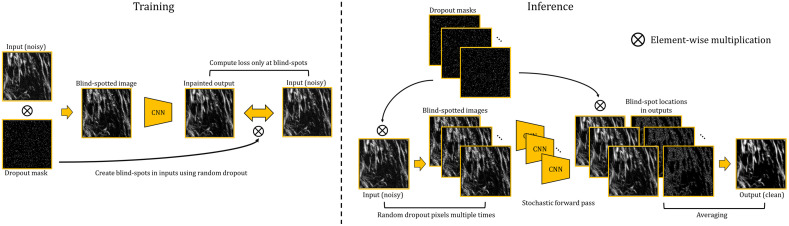
SSD training and inference. During training, blind spots are created throughout the input images. The model is trained to predict the missing values at the blind spots using an image-to-image translation network. Loss is computed only at blind spot locations. At inference, blind spots are created for the input image multiple times, and the outputs at corresponding blind spot locations are averaged to produce the final denoised image.

We used the same idea but modified the original implementation of Noise2Void by making use of random dropout and stochastic forward pass similar to Monte Carlo dropout.^44^ In the original training process of Noise2Void, subregions are randomly cropped from the image. In each subregion, a randomly selected pixel is replaced by another randomly selected pixel. The network is then trained to predict the original pixel value at the replaced location. In the inference, the predictions are then obtained at each location using a sliding window. In our training scheme, we applied random dropout on the input image with a small dropout rate p that creates random blind spots in the input image. The neural network was then trained to predict the missing values at the blind spots. The loss was only evaluated at the blind spot locations to prevent the network from overfitting, which could result in an identity mapping. At inference, a number of k dropout operations with the same dropout rate p were applied to the input image, and k outputs were obtained. Only the pixel values at the blind spot locations in each output were kept. The final output was then computed by averaging the k outputs, similar to the stochastic forward pass used in Bayesian neural networks. We used small values <0.1 for the dropout rate p to ensure that the created blind spots were sparse enough such that each blind spot had enough surrounding pixels observed by the network to achieve a good estimation of the missing pixels. Also the values of k need to be set inversely proportional to p such that the probability of creating a blind spot at each pixel location at least once is close to 1. The training and inference schemes are illustrated in Fig. 4.

#### 2.4.2 Supervised single-image super-resolution model

The goal of SISR models is to estimate the HR image from a given LR image. Let l and h denote an LR image and its corresponding HR image, respectively. The relation between l and h can be written as l=h*f, where * denotes a convolution operation and f denotes the blurring operator. In microscopy, the inverse problem $l*f^{-1}=h$ can be intractable due to the difficulty of finding the true point spread function (blurring operator) and solving the deconvolution itself.^45^ Recently, CNN-based solutions have been proposed to estimate the inverse operation and have achieved state-of-the-art performances.^46^ Most of the CNN-based solutions rely on training on a large amount of LR–HR image pairs, with the difference measurements between the network outputs and ground-truth HR images being minimized. Recently, generative adversarial networks (GANs) have been introduced to enable the CNN to generate HR images with higher visual quality.^47^ Moreover, similar example-based networks can also be applied to the image denoising problem, with the network being trained on noisy-clean image pairs.^48^^,^^49^

**Fig. 5:**
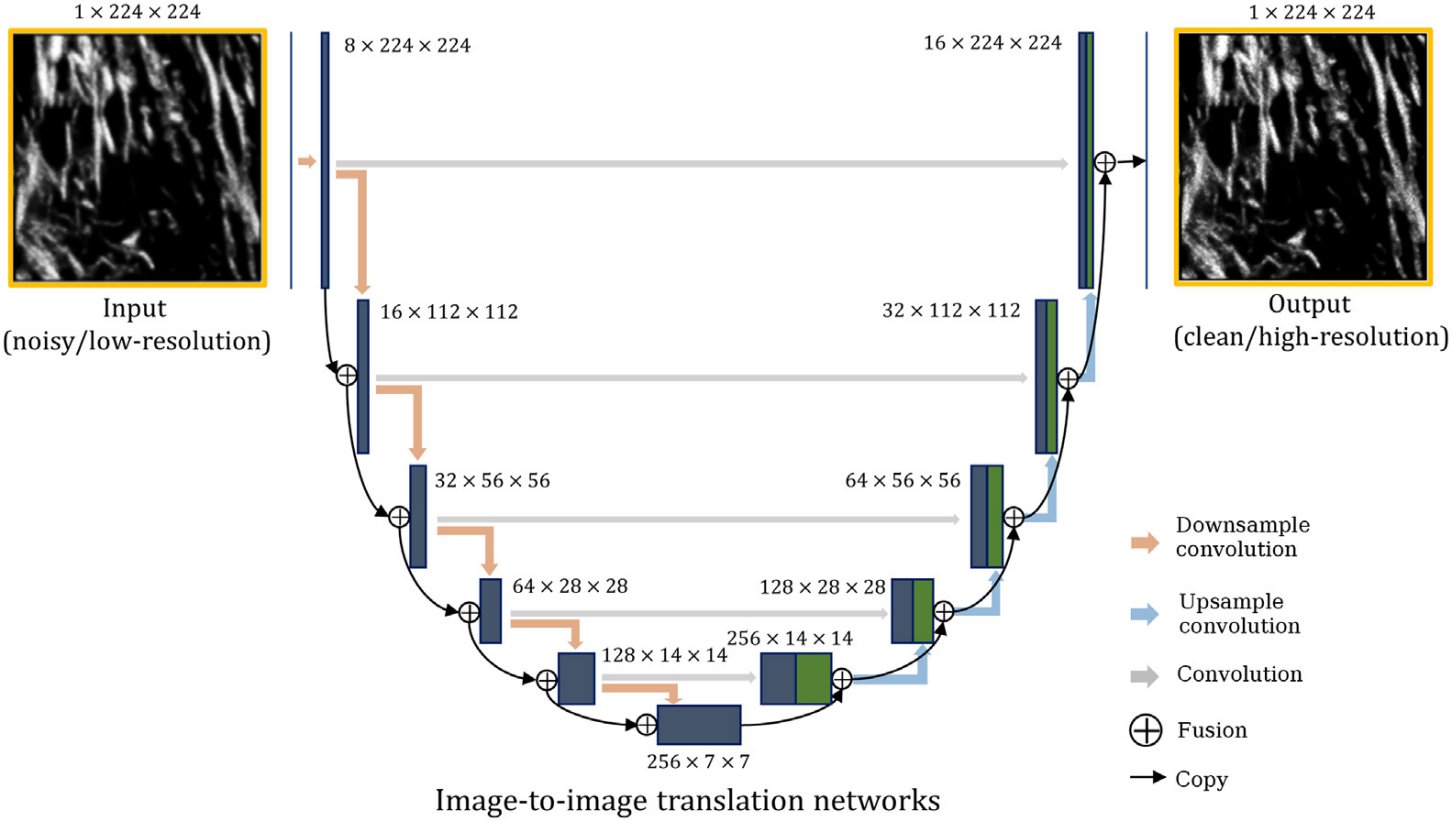
U-Net that maps for image-to-image translation. The network can be trained on image pairs consisting of noisy/clean image pairs for denoising and LR/HR image pairs for training the SISR model. The network can also be used for SSD that uses only noisy images. The expressions in the format of N×N×N denote the number of channels × feature width × feature height for the convolutional layers.

Details of the training specifics can be found in Appendix A.2. The CNN backbone of the models is similar to the image-to-image translation model used in Ref. 50, but with only one input channel (Fig. 5). Although the denoising model can be trained solely using noisy images, the SISR model needs LR/HR image pairs to train. For the SISR model, the input images can be collected at both a low scanning resolution and fast scanning rate (fewer pixels along each dimension and a lower SNR), whereas the “ground truth” images can be acquired at a high scanning resolution with a slow scan rate (more pixels along each dimension and a high SNR). The SISR model can then achieve SISR and denoising simultaneously. Once the models are trained, the user can enable the models during the LSM acquisition for run-time image resolution enhancement and denoising.

To enable the generation of HR and high-SNR images with sharper details and better perceptual quality, we incorporate GANs and perceptual loss for training the image-to-image translation network. GANs have been used in many inverse problems, such as SISR,^51^ image inpainting,^52^ and style transfer.^53^ The discriminator of the implemented GAN consists of five convolutional layers, each followed by a ReLU activation function. The output of the last convolutional layer is processed by an average-pooling layer and fed to a linear layer that produces a single prediction. The discriminator serves as a surrogate to push the generator (the image-to-image translation network) to generate outputs that are indistinguishable by the discriminator.

In addition to GANs, another frequently used approach to increase the visual quality of the generated images is perceptual loss.^54^ Perceptual loss is usually measured by a pretrained CNN in which the features extracted by the CNN are compared between the generated image and the ground truth image. The CNN is pretrained on a third-party dataset (usually ImageNet), and the weights are frozen during the training process of the image-to-image translation network. Thus the features extracted by the CNN can be treated as static latent representations of the input images with structural and semantic meanings. Measuring the distances between the generated image and ground truth image in the latent space provides complementary information that is not available from a pixel-wise loss, such as mean-absolute error (L1 loss) between pair of pixels in the two images. In our implementation, we used ImageNet pretrained VGG16 to extract latent features,^55^ as suggested in Ref. 54. Consider a dataset $D={\left\{x_{i},y_{i}\right\}}_{i=1}^{N}$, where N equals the number of input-target image pairs. The optimization objective of network training is written as

$$G*=\arg\;\underset{G}{\min}\;\frac{1}{N}\overset{N}{\underset{i=1}{\sum }}{\left\|G(x_{i})-y_{i}\right\|}_{1}+\lambda_{p}{\left\|V_{\mathrm{VGG}}(G(x_{i}))-V_{\mathrm{VGG}}(y_{i})\right\|}_{2}+\lambda_{g}\;\arg\;\underset{D}{\max}\;L_{\mathrm{GAN}}(G,D),$$ (3)

$$L_{\mathrm{GAN}}(G,D)=\frac{1}{N}\;\log\;D(y_{i})+\log(1-D(G(x_{i}))),$$ (4)

where G is the generator parameterized by an image-to-image translation network, D is the discriminator described above, and $V_{\mathrm{VGG}}$ is a pretrained VGG16 network. $\lambda_{p}$ is the weight for the perceptual loss, and $\lambda_{g}$ is the weight for the adversarial loss.

**Fig. 6:**
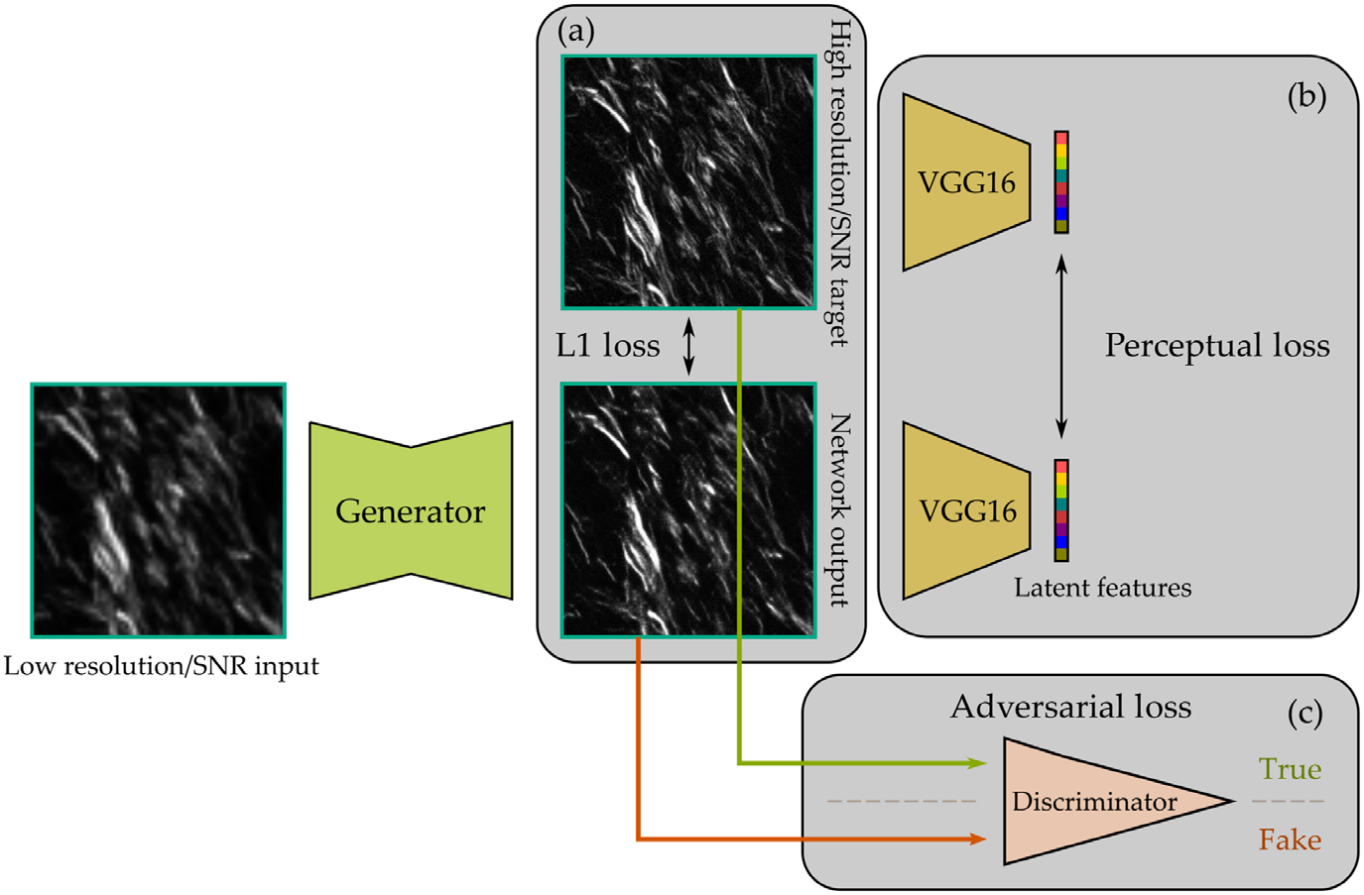
Training procedure of the image-to-image translation network with three types of loss functions. (a) Pixel-wise L1 loss is computed between pixel pairs in the output image and target image. (b) Perceptual loss is measured as the mean-square error between the latent features extracted by a pretrained VGG16 of the output image and target image. (c) A discriminator is trained on par with the generator and pushes the generator to generate outputs that are hard to be distinguished by the discriminator.

The resulting training pipeline that optimizes all three kinds of loss functions is illustrated in Fig. 6. Similar concepts have been implemented for autofluorescence-harmonic microscopy.^56^ Our major contribution lies in integrating the methods into a run-time analysis workflow along with the acquisition. This system would also facilitate the collection of paired image data of different resolutions and different modalities for training such DL models. We evaluated the efficacy of the image-to-image translation network for denoising and resolution enhancement of SHG images, with results presented in the next section.

## 3 Experiments and Results

### 3.1 Samples and Datasets

The samples used for imaging were eight TMA slides bought from US Biomax,^57^ BBS14011, PA2072, PA961e, PA485, PA802, PA2081b, HPan-Ade120Sur, and Hpan-Ade170Sur.

The TMAs contain a total of 769 TMA cores of pancreatic cancer (pancreatic ductal adenocarcinoma), chronic pancreatitis, and normal pancreas tissue. Cores were generally 2 mm in diameter and 4-μm thick. PA2081b, containing a mixture of the three types of cores, was used to collect data with annotations generated automatically by the machine learning detector.

This TMA slide was also used for testing the run-time LSM image denoising and resolution enhance models. The rest of the slides were used for collecting BF and SHG data from manually drawn annotations and the data were used to train the DNNs. Further details regarding each TMA slide can be found in Ref. 57. The hardware of the machine used to control the imaging system is as follows: CPU: Intel^®^ Core™ i9-10900X CPU 3.70 GHz (10 cores), RAM: 32 GB, GPU: NVIDIA RTX A4000 (16 GB).

#### 3.1.1 Datasets for machine learning tumor region detector

TMA cores were separated into two categories, 459 malignant (pancreatic cancer) and 310 benign cores (chronic pancreatitis and normal pancreas tissue). The BF images captured at 4× were used for training and validation after being divided into patches with a size of 224×224 without overlap.

Low saturation patches (mean saturation <0.15) were discarded to exclude background patches, with saturation defined as per the hue, saturation, brightness color model. 80% of the cores (18,631 patches) were used for training, and 20% of the cores (4658 patches) were used for validation.

#### 3.1.2 Datasets for run-time image restoration

Slides BBS14011, PA2072, and PA961e were used to collect BF images and SHG images at 20×.

In all cases, the 20× BF fields of view were 230.9  μm×309.0  μm, and the SHG FOVs were 130.6  μm×130.6  μm.

SHG images were collected using two settings. (1)  Low-quality setting: scan rate at 500,000 Hz, scanning resolution at 256×256 (pixel size 0.509  μm per pixel). (2) High-quality setting: scan rate at 100,000 Hz, scanning resolution at 512×512 (pixel size 0.255  μm per pixel). The three TMA slides yielded 28,464 tiles, each containing a z-stack of five slices, excluding low signal tiles (mean pixel value <0.1). 20% of the tiles were split for validation during the training to prevent overfitting. The trained network was then used to perform prediction on the testing TMA slide (PA2081b) as a part of the automatic acquisition workflow. The z-stack step size was 4  μm.

The total z-stack range covered small variations in the tissue surface. In training, each slice of a z-stack tile was treated as an input image.

### 3.2 Selective Acquisition in Multiscale and Multimodal

#### 3.2.1 Manual annotation

**Fig. 7:**
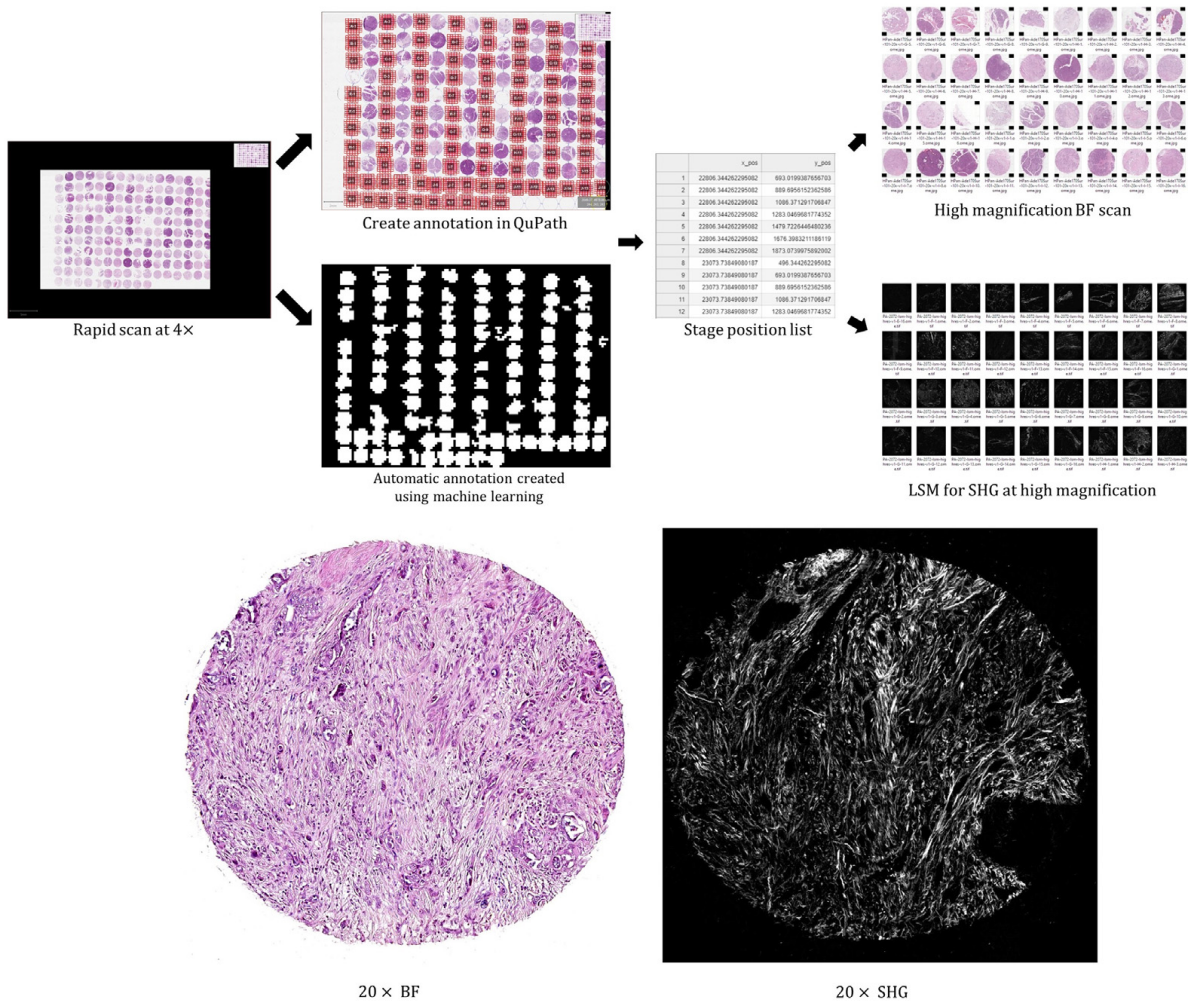
Selective acquisition using our system. A rapid BF scan is first conducted at 4×. Annotations are then created manually in QuPath or using the machine learning model trained for target detection. The annotations are then converted into position list files to be read by the acquisition program for BF and SHG acquisition at 20×. The scale bar for the bottom images is 200  μm.

Once the slide is mounted on the stage, the rapid 4× scan function is executed. Image tiles are automatically white-balanced, flat-field corrected, stitched, and exported as a pyramidal OME.TIFF^33^^,^^58^ files. To make annotations manually, the user opens the 4× image in QuPath and draw annotations with QuPath annotation tools. Annotations with arbitrary closed shapes are supported. The system uses a set of QuPath scripts (written in Groovy) that can convert the annotations into stage position lists that can be read by the acquisition program. The position list is automatically passed to the acquisition program when the 20× and SHG acquisition workflows are executed. Multiple position lists are generated and executed in a loop for multiple annotations on a slide. The acquisition procedure and results of this functionality are shown in Fig. 7.

#### 3.2.2 Automatic annotation by machine learning

Once the rapid scan at 4× finishes, the tiles can be examined by a machine learning model. For the training of the supervised learning model, all patches extracted from a malignant core were considered positive, and all patches from a benign core were considered negative. The classification scores of the patches were averaged for each core to reach a core-level prediction. After training, the supervised model achieved an accuracy of 94.5% in differentiating pancreatic cancer cores from benign pancreas cores in the validation set. At acquisition, the model processes the tiles, makes predictions on the nonempty tiles (mean saturation >0.15), and generates a position list containing the locations of the positive areas. The position list is then imported to QuPath and displayed as annotations by executing a QuPath script. The detection model successfully classified 175 cores (out of 192 cores) on the testing TMA, with an accuracy of 91.1% and a recall of 0.942. The downstream acquisition at 20× for BF and SHG were then performed using the position list, as shown in Fig. 7.

### 3.3 Run-Time Image Restoration

The trained denoising model and resolution enhancement model are enabled during the acquisition by specifying them as the image processing hook function in the Pycro-Anager Acquisition API. The image processing hook function provides access to the data-stream as data is being acquired. This allows the data to be modified, analyzed, and diverted to customized visualization and saving on-the-fly. For evaluating the SSD model and SISR model, the models are enabled with a fast scanning pixel rate of 0.5 MHz at a 256×256 scanning resolution. The processed images are compared with the ground truth images collected using a slow scanning pixel rate of 0.1 MHz at a 512×512 scanning resolution. Note that the same scanning rates and scanning resolutions are used to collect the training data for model training. The SSD model is trained using SHG images collected at 0.5 MHz at a 256×256 scanning resolution, whereas the SISR model is trained to map SHG images collected at 0.1 MHz at a 256×256 scanning resolution to images collected at 0.1 MHz at a 512×512 scanning resolution.

Four metrics are used to evaluate the similarity between the network outputs and the ground truth: peak-signal-to-noise ratio (PSNR), structural similarity (SSIM),^59^ Fréchet inception distance (FID),^60^ and CurveAlign collagen fiber statistics.^61^ PSNR is a pixel-wise metric that measures the pair-wise distances of pixels in the same locations of two images. SSIM and FID are perception-based metrics that quantify the visual similarity between images based on measurements derived from the whole images or regions instead of individual pixels. SSIM follows an explicitly defined formula, whereas FID is based on features computed from pretrained Inception networks.^62^ FID is shown to have the ability to produce measurements that align well with visual observations of human, and it is currently the standard metric to assess the quality of images generated by GANs.^63^

CurveAlign is a popular tool box for characterizing collagen fiber topography, such as measuring collagen fiber density, and fiber alignment coefficients.^61^ We computed the absolute differences between the collagen fiber alignment coefficient and collagen fiber density calculated by CurveAlign between the processed image and ground truth image. The absolute difference for each image is than divided by the maximum value of alignment coefficient and density in the testing set, respectively, resulting in absolute error ratios for alignment coefficient and density. The ratios are then averaged across the testing set. This metric can be seen as another type of image-level metric with collagen-specific domain knowledge, selected due to collagen structure being the biologically relevant focus of the SHG imaging.

**Fig. 8:**
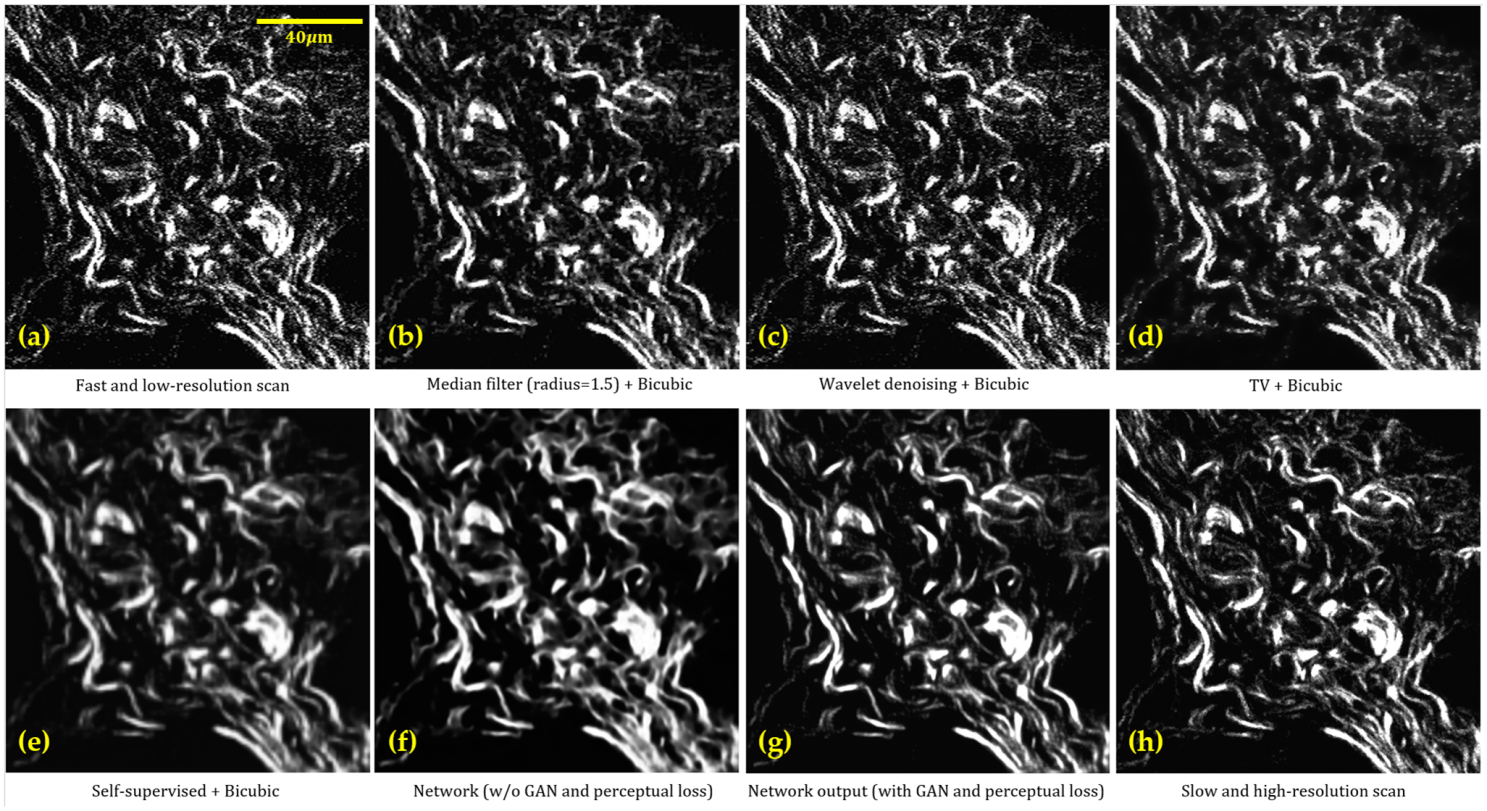
Denoising of SHG images: (a) noisy and LR image collected using a faster scanning rate (0.5 MHz) at a 256×256 scanning resolution; (b) median filter (radius = 1.5) followed by bicubic upscaling; (c) wavelet-based denoising followed by bicubic upscaling; (d) TV-based denoising followed by bicubic upscaling; (e) SSD followed by bicubic upscaling; (f) supervised denoising using the SISR network without GAN and perceptual loss; (g) supervised denoising using the SISR network with GAN and perceptual loss; and (h) clean and HR image collected using a slow scanning rate (0.1 MHz) at a 512×512 scanning resolution.

The outputs of the supervised method (SISR network) and self-supervised method are compared with several traditional denoising methods, including median filter, wavelet-based denoising,^64^ and total variation (TV)-based denoising.^65^ After performing denoising, the resulting images are upscaled using bicubic interpolation to match the size of the ground truth images collected using a slower scanning rate and a higher scanning resolution. Note that the SISR network performs denoising and upscaling simultaneously in a single network and bicubic upscaling is not needed. Details of the quantitative evaluation of the outputs are summarized in Table 1. Some representative outputs generated by the baselines and DL-based methods are shown in Fig. 8.

**Table 1:** Comparison of different denoising and upscaling methods.

	PSNR (dB)	SSIM	FID	Alignment error (%)	Density error
Bicubic	20.97	0.401	110.1	37.4	12.2
Median + bicubic	24.88	0.518	61.1	23.0	7.8
Wavelet + bicubic	21.10	0.409	80.9	37.7	11.0
TV + bicubic	23.03	0.501	86.4	27.4	6.6
Self-supervised + bicubic	25.71	0.623	84.3	23.7	5.9
SISR w/o GAN and perceptual loss	28.34	0.831	69.7	21.8	3.4
SISR with GAN and perceptual loss	27.90	0.835	37.6	15.5	4.0

Bicubic: use bicubic upsampling on the noisy images. Median + bicubic: use median filter (radius = 1.5) on the noisy images and upsample the outputs using bicubic upsampling. Wavelet + Bicubic: use wavelet-based denoising on the noisy images and upsample the outputs using bicubic upsampling. TV + bicubic: use TV-based denoising on the noisy images and upsample the outputs using bicubic upsampling. Self-supervised + bicubic: upsample the outputs of the SSD network using bicubic upsampling. SISR w/o GAN and perceptual loss: the outputs of the SISR network without using GAN and perceptual loss. SISR with GAN and perceptual loss: the outputs of the SISR network with GAN and perceptual loss enabled during the training. For PSNR and SSIM, larger values suggest a higher similarity between the outputs and the ground truth. SSIM has a value range from 0 to 1, and the value reaches 1 if two images are identical. For FID, alignment error, and density error, smaller values suggest a higher similarity between the outputs and the ground truth.

The evaluation results show that for SHG image denoising, SSD compares favorably to traditional methods. For the SISR model, the network processed images better resemble the ground truth images with better PSNR, SSIM, and FID score and more similar collagen fiber alignment coefficient and density. The results also suggest that the addition of GAN and perceptual loss increases the visual quality of the generated images, with a smaller FID score and a higher SSIM.

The computation time of the SISR model is around 0.2  s/frame, and the scanning time for each frame is reduced from ∼3 to ∼0.15  s (a breakdown of run times can be found in Appendix C, Table 2). The superior performance in image restoration quality and the resulting acquisition acceleration demonstrate the high efficacy of DL-based methods for run-time LSM image processing.

## 4 Conclusion and Future Work

In this paper, we presented a hardware–software co-design for automatic and reproducible multimodal imaging of histological slides. The system integrates the acquisition and analysis in a single workflow that minimizes human intervention and improves the throughput for complex imaging experiments. The Python-based Pycro-Manager bridges the gap between the Python environment and the Java-based open-source microscope control software, Micro-Manager. Thus the controlling program accesses the existing Java libraries to communicate with the hardware device adaptors, including the laser scanning module OpenScan, that drives the microscope hardware, while making use of a large number of Python data analysis packages for building DL models and performing image processing. Together with the coupled BF and LSM light path, our system can switch between modalities and reach optimized configuration presets for each modality and magnification automatically by running Python scripts. QuPath is used for data visualization and manual annotations, after which the stage coordinates computed from the annotations are transferred back into the acquisition program for downstream selective acquisition. The DL-based detection model used in our system can automatically generate annotations for targets in the specimen and switch to point scanning mode for the targeted area. Moreover, our system benefits from run-time image enhancement for LSM that can improve the image SNR and image resolution, leading to shorter scan times while maintaining the necessary image quality for downstream analysis to answer biological questions.

It should be noted that such image enhancement has limits, which depend on the details of the modality and the hardware of the system in question. A lower numerical aperture 4× objective, for example, might not generate sufficient information to accurately enhance an image to a final apparent magnification of 20× when performing super-resoution. Such limits should be testable by comparing HR images with paired restored LR counterparts.

It is also worthwhile mentioning that restoring clean/HR images from their noisy/LR counterparts is an under-determined problem, meaning that, for the input (e.g., an LR image), there exist multiple possible outputs (e.g., HR images that can be downsampled to match the same LR image). In this case, AI-generated outputs can be prone to blurriness because the model will tend to output an averaged result of all possible results. This effect will become more prominent as the resolution gap between the input and target output increases because there will be more possible HR outputs. Although adversarial loss and perceptual loss are useful for fighting off this effect to some extent,^1^ this effect cannot be completely eliminated and sometimes can cause instability for adversarial network training due to the nature of under-determined problems.

Future work includes improving the speed of software autofocus by developing a DL-based one-shot autofocus method^66^ for BF imaging and implementing support and denoising models for FLIM, an additional modality that is already supported by Micro-Manager and OpenScan.

Additionally, adapting this work to other system hardware, other systems, and other stains and tissue types would allow for an analysis of how generalizable the method is and how much additional data and training time will be necessary to apply these DL models to new data. For optimization, the workflow would need to integrate training data from the new system and adapt the existing model to the new system.

## 5 Appendix A: Deep Learning Model Training Details

### 5.1 Detection Model

The CNN backbone used to train the detection models is ResNet18.^41^ The optimizer is Adam^67^ with a learning rate of 0.0002 and cosine annealing learning scheduler^68^ without warm restart. The batch size for the supervised model is 64. The loss function is cross-entropy loss and binary cross-entropy loss, respectively. The number of training epochs is 100.

### 5.2 Runtime Image Enhancement Model

The image-to-image translation CNN backbone is described in Ref. 50, without the discriminator network, with the input and output channel being set to 1 for grayscale LSM images. The optimizer is Adam^67^ with a learning rate of 0.0002 and cosine annealing learning scheduler^68^ without warm restart. The batch size is 32 and the loss function is mean square error. The number of training epoch is 100. The input dropout rate is p=0.1 and the number of passes of the stochastic forward pass at inference is 1/p×64.

## 6 Appendix B: Software Autofocus Algorithm

The software autofocus algorithm (Algorithm 1) that runs during brightfield image acquisitions of tissue sections:

**Algorithm 1:** Software autofocus.

Move z-stage to previous returned **z position**;
Generate 5 **z positions** with an equal interval symmetric to the current **z position**;
for ***idx*** *in* ***z*** **positions do**
Move z-stage to **idx**;
Capture an image;
Apply canny filter to the image;
Sum the filtering result to produce a focus score;
Store the focus score in array A;
**End**
Apply cubic interpolation to A and obtain the upsampled array;
Find the corresponding **z position** value with the maximum focus score in A;
**Return** **z position**

## 7 Appendix C: Computation Hardware and Run Time Breakdown

The training of the tumor region detection network involves 769 TMA cores (23,289 patches at 4×) and takes roughly 5 h for training on an RTX 2080 Ti GPU. The training of the SHG image enhancement network involves 351 TMA cores (142,320 patches at 20× with z slices) and takes roughly 30 h for training on an RTX 2080 Ti GPU with a host PC running an Intel i9-10800x CPU. Multimodal whole slide scanning times for a workflow with run-time image enhancement at a 256×256 scanning resolution and 500,000 Hz scanning rate and without run-time image enhancement at a 512×512 scanning resolution and 100,000 Hz on the testing TMA slide (PA2081b, 192 cores, 16,682 tiles) are shown in Table 2.

**Table 2:** Acquisition times for the components in the workflows with and without run-time image enhancement.

Method	SHG tile scanning time	SHG slide scanning time (h)	4× BF scanning time (h)	20× BF scanning time (h)	Total time (h)
Baseline	3.57 s × 5	97.6	0.08	1.1	98.78
Enhanced	0.43 s × 5	11.8	0.08	1.1	12.98

## Code, Data, and Materials Availability

The GitHub repository of the project is at https://github.com/uw-loci/smart-wsi-scanner/tree/master (acquisition program) and https://github.com/uw-loci/lsm-run-time-enhancement (deep learning models).

## References

[1] Cornish T. C. Swapp R. E. Kaplan K. J., “Whole-slide imaging: routine pathologic diagnosis,” Adv. Anatomic Pathol. 19(3), 152–159 (2012). 10.1097/PAP.0b013e318253459e 22498580

[2] Pantanowitz L. et al., “Review of the current state of whole slide imaging in pathology,” J. Pathol. Inf. 2(1), 36 (2011). 10.4103/2153-3539.83746 PMC3162745 21886892

[3] Gurcan M. N. et al., “Histopathological image analysis: a review,” IEEE Rev. Biomed. Eng. 2, 147–171 (2009). 10.1109/RBME.2009.2034865 20671804 PMC2910932

[4] Ghaznavi F. et al., “Digital imaging in pathology: whole-slide imaging and beyond,” Annu. Rev. Pathol. Mech. Dis. 8, 331–359 (2013). 10.1146/annurev-pathol-011811-120902 23157334

[5] Litjens G. et al., “Deep learning as a tool for increased accuracy and efficiency of histopathological diagnosis,” Sci. Rep. 6, 26286 (2016). SRCEC3 2045-2322 10.1038/srep26286 27212078 PMC4876324

[6] Srinidhi C. L. Ciga O. Martel A. L., “Deep neural network models for computational histopathology: a survey,” Med. Image Anal. 67, 101813 (2021). 10.1016/j.media.2020.101813 33049577 PMC7725956

[7] Lichtman J. W. Conchello J.-A., “Fluorescence microscopy,” Nat. Methods 2(12), 910–919 (2005).1548-7091 10.1038/nmeth817 16299476

[8] Larson A. M., “Multiphoton microscopy,” Nat. Photonics 5, 1 (2011). NPAHBY 1749-4885 10.1038/nphoton.an.2010.2

[9] Chen X. et al., “Second harmonic generation microscopy for quantitative analysis of collagen fibrillar structure,” Nat. Protoc. 7(4), 654–669 (2012).1754-2189 10.1038/nprot.2012.009 22402635 PMC4337962

[10] Keikhosravi A. et al., “Quantification of collagen organization in histopathology samples using liquid crystal based polarization microscopy,” Biomed. Opt. Express 8(9), 4243–4256 (2017). BOEICL 2156-7085 10.1364/BOE.8.004243 28966862 PMC5611938

[r11] Majeed H. et al., “Quantitative phase imaging for medical diagnosis,” J. Biophotonics 10(2), 177–205 (2017). 10.1002/jbio.201600113 27539534

[r12] Wang Z. et al., “Spatial light interference microscopy (SLIM),” Opt. Express 19(2), 1016–1026 (2011). OPEXFF 1094-4087 10.1364/OE.19.001016 21263640 PMC3482902

[13] Keikhosravi A. et al., “Real-time polarization microscopy of fibrillar collagen in histopathology,” Sci. Rep. 11, 19063 (2021). SRCEC3 2045-2322 10.1038/s41598-021-98600-w 34561546 PMC8463693

[14] Cicerone M. T. Camp C. H., “Histological coherent Raman imaging: a prognostic review,” Analyst 143(1), 33–59 (2018). ANLYAG 0365-4885 10.1039/C7AN01266G 29098226

[15] Fernandez D. C. et al., “Infrared spectroscopic imaging for histopathologic recognition,” Nat. Biotechnol. 23(4), 469–474 (2005). NABIF9 1087-0156 10.1038/nbt1080 15793574

[16] Conklin M. W. et al., “Aligned collagen is a prognostic signature for survival in human breast carcinoma,” Am. J. Pathol. 178(3), 1221–1232 (2011). AJPAA4 0002-9440 10.1016/j.ajpath.2010.11.076 21356373 PMC3070581

[17] Provenzano P. P. et al., “Collagen reorganization at the tumor-stromal interface facilitates local invasion,” BMC Med. 4(1), 1–15 (2006). 10.1186/1741-7015-4-38 17190588 PMC1781458

[18] Datta R. et al., “Fluorescence lifetime imaging microscopy: fundamentals and advances in instrumentation, analysis, and applications,” J. Biomed. Opt. 25(7), 071203 (2020). JBOPFO 1083-3668 10.1117/1.JBO.25.7.071203 32406215 PMC7219965

[19] Chacko J. V. Eliceiri K. W., “NAD(P)H fluorescence lifetime measurements in fixed biological tissues,” Methods Appl. Fluoresc. 7(4), 044005 (2019). 10.1088/2050-6120/ab47e5 31553966

[20] Migliozzi D. et al., “Multimodal imaging and high-throughput image-processing for drug screening on living organisms on-chip,” J. Biomed. Opt. 24(2), 021205 (2018). JBOPFO 1083-3668 10.1117/1.JBO.24.2.021205 30484295 PMC6987638

[r21] Kwak J. T. et al., “Multimodal microscopy for automated histologic analysis of prostate cancer,” BMC Cancer 11(1), 1–16 (2011). BCMACL 1471-2407 10.1186/1471-2407-11-62 21303560 PMC3045985

[22] Schlichenmeyer T. C. et al., “Video-rate structured illumination microscopy for high-throughput imaging of large tissue areas,” Biomed. Opt. Express 5(2), 366–377 (2014). BOEICL 2156-7085 10.1364/BOE.5.000366 24575333 PMC3920869

[23] Bankhead P. et al., “Qupath: open source software for digital pathology image analysis,” Sci. Rep. 7, 16878 (2017). SRCEC3 2045-2322 10.1038/s41598-017-17204-5 29203879 PMC5715110

[24] Marée R. et al., “Collaborative analysis of multi-gigapixel imaging data using cytomine,” Bioinformatics 32(9), 1395–1401 (2016). BOINFP 1367-4803 10.1093/bioinformatics/btw013 26755625 PMC4848407

[25] Gutman D. A. et al., “The digital slide archive: a software platform for management, integration, and analysis of histology for cancer research,” Cancer Res. 77(21), e75–e78 (2017). CNREA8 0008-5472 10.1158/0008-5472.CAN-17-0629 29092945 PMC5898232

[26] Schindelin J. et al., “Fiji: an open-source platform for biological-image analysis,” Nat. Methods 9(7), 676–682 (2012).1548-7091 10.1038/nmeth.2019 22743772 PMC3855844

[27] Carpenter A. E. et al., “Cellprofiler: image analysis software for identifying and quantifying cell phenotypes,” Genome Biol. 7(10), 1–11 (2006). GNBLFW 1465-6906 10.1186/gb-2006-7-10-r100 PMC1794559 17076895

[28] Edelstein A. D. et al., “Advanced methods of microscope control using μ manager software,” J. Biol. Methods 1(2), e10 (2014). 10.14440/jbm.2014.36 25606571 PMC4297649

[29] Conrad C. et al., “Micropilot: automation of fluorescence microscopy-based imaging for systems biology,” Nat. Methods 8(3), 246–249 (2011).1548-7091 10.1038/nmeth.1558 21258339 PMC3086017

[30] Dietz C. Berthold M. R., “Knime for open-source bioimage analysis: a tutorial,” Adv. Anat. Embryol. Cell Biol. 219, 179–197 (2016). AAEBDS 0301-5556 10.1007/978-3-319-28549-8_7 27207367

[31] Pinkard H. et al., “Pycro-manager: open-source software for customized and reproducible microscope control,” Nat. Methods 18(3), 226–228 (2021).1548-7091 10.1038/s41592-021-01087-6 33674797 PMC8532176

[32] Goode A. et al., “Openslide: a vendor-neutral software foundation for digital pathology,” J. Pathol. Inf. 4, 27 (2013). 10.4103/2153-3539.119005 PMC3815078 24244884

[33] Besson S. et al., “Bringing open data to whole slide imaging,” Lect. Notes Comput. Sci. 11435, 3–10 (2019). LNCSD9 0302-9743 10.1007/978-3-030-23937-4_1 PMC6774793 31579322

[34] Preibisch S. Saalfeld S. Tomancak P., “Globally optimal stitching of tiled 3D microscopic image acquisitions,” Bioinformatics 25(11), 1463–1465 (2009). BOINFP 1367-4803 10.1093/bioinformatics/btp184 19346324 PMC2682522

[35] Dai B. et al., “OpenScan,” https://eliceirilab.org/openscan/ (accessed 26 January 2023).

[36] Chen P.-H. C. et al., “An augmented reality microscope with real-time artificial intelligence integration for cancer diagnosis,” Nat. Med. 25(9), 1453–1457 (2019).1078-8956 10.1038/s41591-019-0539-7 31406351

[r37] Campanella G. et al., “Clinical-grade computational pathology using weakly supervised deep learning on whole slide images,” Nat. Med. 25(8), 1301–1309 (2019).1078-8956 10.1038/s41591-019-0508-1 31308507 PMC7418463

[r38] Li B. Li Y. Eliceiri K. W., “Dual-stream multiple instance learning network for whole slide image classification with self-supervised contrastive learning,” in Proc. IEEE/CVF Conf. Comput. Vis. And Pattern Recognit., pp. 14318–14328 (2021). 10.1109/CVPR46437.2021.01409 PMC8765709 35047230

[39] Sirinukunwattana K. et al., “Gland segmentation in colon histology images: the glas challenge contest,” Med. Image Anal. 35, 489–502 (2017). 10.1016/j.media.2016.08.008 27614792

[40] Lowe D. G., “Distinctive image features from scale-invariant keypoints,” Int. J. Comput. Vis. 60(2), 91–110 (2004). IJCVEQ 0920-5691 10.1023/B:VISI.0000029664.99615.94

[41] He K. et al., “Deep residual learning for image recognition,” in Proc. IEEE Conf. Comput. Vis. And Pattern Recognit., pp. 770–778 (2016). 10.1109/CVPR.2016.90

[42] Krull A. Buchholz T.-O. Jug F., “Noise2void-learning denoising from single noisy images,” in Proc. IEEE/CVF Conf. Comput. Vis. And Pattern Recognit., pp. 2129–2137 (2019). 10.1109/CVPR.2019.00223

[43] Laine S. et al., “High-quality self-supervised deep image denoising,” in Adv. In Neural Inf. Process. Syst., Vol. 32 (2019).

[44] Gal Y. Ghahramani Z., “Dropout as a Bayesian approximation: representing model uncertainty in deep learning,” in Int. Conf. Mach. Learn., PMLR, pp. 1050–1059 (2016).

[45] Freeman W. T. Jones T. R. Pasztor E. C., “Example-based super-resolution,” IEEE Comput. Graph. Appl. 22(2), 56–65 (2002). ICGADZ 0272-1716 10.1109/38.988747

[46] Dong C. et al., “Image super-resolution using deep convolutional networks,” IEEE Trans. Pattern Anal. Mach. Intell. 38(2), 295–307 (2016). ITPIDJ 0162-8828 10.1109/TPAMI.2015.2439281 26761735

[47] Ledig C. et al., “Photo-realistic single image super-resolution using a generative adversarial network,” in Proc. IEEE Conf. Comput. Vis. And Pattern Recognit., pp. 4681–4690 (2017). 10.1109/CVPR.2017.19

[48] Zhang K. et al., “Beyond a Gaussian denoiser: residual learning of deep CNN for image denoising,” IEEE Trans. Image Process. 26(7), 3142–3155 (2017). IIPRE4 1057-7149 10.1109/TIP.2017.2662206 28166495

[49] Jain V. Seung S., “Natural image denoising with convolutional networks,” in Adv. In Neural Inf. Process. Syst., Vol. 21 (2008).

[50] Li B. et al., “Single image super-resolution for whole slide image using convolutional neural networks and self-supervised color normalization,” Med. Image Anal. 68, 101938 (2021). 10.1016/j.media.2020.101938 33359932

[51] Ledig C. et al., “Photo-realistic single image super-resolution using a generative adversarial network,” in IEEE Conf. Comput. Vis. And Pattern Recognit. (CVPR), IEEE, pp. 105–114 (2017). 10.1109/CVPR.2017.19

[52] Isola P. et al., “Image-to-image translation with conditional adversarial networks,” in IEEE Conf. Comput. Vis. And Pattern Recognit. (CVPR), IEEE, Honolulu, HI, pp. 5967–5976 (2017). 10.1109/CVPR.2017.632

[53] Zhu J.-Y. et al., “Unpaired image-to-image translation using cycle-consistent adversarial networks,” in IEEE Int. Conf. Comput. Vis. (ICCV), IEEE, Venice, pp. 2242–2251 (2017). 10.1109/ICCV.2017.244

[54] Johnson J. Alahi A. Fei-Fei L., “Perceptual losses for real-time style transfer and super-resolution,” Lect. Notes Comput. Sci. 9906, 694–711 (2016). LNCSD9 0302-9743 10.1007/978-3-319-46475-6_43

[55] Simonyan K. Zisserman A., “Very deep convolutional networks for large-scale image recognition,” arXiv:1409.1556 (2014).

[56] Shen B. et al., “Deep learning autofluorescence-harmonic microscopy,” Light Sci. Appl. 11(1), 1–14 (2022). 10.1038/s41377-022-00768-x 35351853 PMC8964717

[57] TissueArray.Com LLC, “TissueArray.Com (USBiomax),” https://www.tissuearray.com (accessed 26 January 2023).

[58] Swedlow J. R. et al., “Bioimage informatics for experimental biology,” Annu. Rev. Biophys. 38, 327 (2009). ARBNCV 1936-122X 10.1146/annurev.biophys.050708.133641 19416072 PMC3522875

[59] Wang Z. et al., “Image quality assessment: from error visibility to structural similarity,” IEEE Trans. Image Process. 13(4), 600–612 (2004). IIPRE4 1057-7149 10.1109/TIP.2003.819861 15376593

[60] Heusel M. et al., “Gans trained by a two time-scale update rule converge to a local nash equilibrium,” in Adv. In Neural Inf. Process. Syst., Vol. 30 (2017).

[61] Liu Y. et al., “Methods for quantifying fibrillar collagen alignment,” in Fibrosis, Rittié L., ed., pp. 429–451, Springer (2017). 10.1007/978-1-4939-7113-8_28 PMC6343484 28836218

[62] Szegedy C. et al., “Rethinking the inception architecture for computer vision,” in Proc. IEEE Conf. Comput. Vis. And Pattern Recognit., pp. 2818–2826 (2016). 10.1109/CVPR.2016.308

[63] Lucic M. et al., “Are gans created equal? A large-scale study,” in Adv. In Neural Inf. Process. Syst., Vol. 31 (2018).

[64] Chang S. G. Yu B. Vetterli M., “Adaptive wavelet thresholding for image denoising and compression,” IEEE Trans. Image Process. 9(9), 1532–1546 (2000). IIPRE4 1057-7149 10.1109/83.862633 18262991

[65] Chambolle A., “An algorithm for total variation minimization and applications,” J. Math. Imaging Vis. 20(1), 89–97 (2004). 10.1023/B:JMIV.0000011325.36760.1e

[66] Pinkard H. et al., “Deep learning for single-shot autofocus microscopy,” Optica 6(6), 794–797 (2019). 10.1364/OPTICA.6.000794

[67] Kingma D. P. Ba J., “Adam: a method for stochastic optimization,” arXiv:1412.6980 (2014).

[68] Loshchilov I. Hutter F., “SGDR: stochastic gradient descent with warm restarts,” arXiv:1608.03983 (2016).

